# Role of Oxidative Stress in Heart Failure: Insights from Gene Transfer Studies

**DOI:** 10.3390/biomedicines9111645

**Published:** 2021-11-09

**Authors:** Bart De Geest, Mudit Mishra

**Affiliations:** 1 Centre for Molecular and Vascular Biology, Catholic University of Leuven, 3000 Leuven, Belgium; 2 Department of Cardiothoracic Surgery, University Medical Center Utrecht, 3584 CX Utrecht, The Netherlands; drmuditm@gmail.com

**Keywords:** gene therapy, gene transfer, heart failure, oxidative stress, reactive oxygen species, cardiac hypertrophy, cardiac remodeling, microRNA

## Abstract

Under physiological circumstances, there is an exquisite balance between reactive oxygen species (ROS) production and ROS degradation, resulting in low steady-state ROS levels. ROS participate in normal cellular function and in cellular homeostasis. Oxidative stress is the state of a transient or a persistent increase of steady-state ROS levels leading to disturbed signaling pathways and oxidative modification of cellular constituents. It is a key pathophysiological player in pathological hypertrophy, pathological remodeling, and the development and progression of heart failure. The heart is the metabolically most active organ and is characterized by the highest content of mitochondria of any tissue. Mitochondria are the main source of ROS in the myocardium. The causal role of oxidative stress in heart failure is highlighted by gene transfer studies of three primary antioxidant enzymes, thioredoxin, and heme oxygenase-1, and is further supported by gene therapy studies directed at correcting oxidative stress linked to metabolic risk factors. Moreover, gene transfer studies have demonstrated that redox-sensitive microRNAs constitute potential therapeutic targets for the treatment of heart failure. In conclusion, gene therapy studies have provided strong corroborative evidence for a key role of oxidative stress in pathological remodeling and in the development of heart failure.

## 1. Introduction

### 1.1. General Aim of the Review

Oxidative stress is assumed to play a key role in pathological cardiac hypertrophy and remodeling and in the development of heart failure. Gene transfer intervention studies provide a very suitable tool to prove the causal role of oxidative stress in these processes. Following an introduction on the physiological and pathophysiological role of reactive oxygen species (ROS) in the heart, we will analyze the causal role of oxidative stress in heart failure from the angle of gene transfer studies of the three primary antioxidant enzymes (superoxide dismutase (SOD), catalase, and glutathione peroxidase), of thioredoxin, and of heme oxygenase-1.

Secondly, we will review the converging evidence from gene transfer studies demonstrating that gene therapy directed at correction of metabolic risk factors results in a marked reduction of systemic oxidative stress and of oxidative stress in the myocardium, in an improved cardiac function, and in prevention or reversal of pathological remodeling and heart failure. Thirdly, we will discuss gene transfer of microRNAs and modulation of microRNA activity to reduce oxidative stress and to prevent heart failure. Taken together, the general aim of this paper is to review this field both from a therapeutic perspective and from the perspective of demonstrating the causal role of oxidative stress in pathological hypertrophy, cardiac dysfunction, and heart failure.

### 1.2. Reactive Oxygen Species

ROS are defined based on their higher reactivity than molecular oxygen (O_2_). The intermediates of successive one-electron reductions of molecular oxygen are superoxide anion radical (O_2_^.−^), hydrogen peroxide (H_2_O_2_), and hydroxyl radical (HO^.^). All electrons in hydrogen peroxide are paired, and this molecule is therefore not a free radical. Nevertheless, hydrogen peroxide has a higher chemical reactivity than molecular oxygen and therefore belongs to the ROS. ROS also include peroxyl (RO_2_^.^), hydroperoxyl (HO_2_^.^), alkoxyl (RO^.^), peroxyl (ROO^.^), nitric oxide (NO^.^), nitrogen dioxide (NO_2_^.^), lipid peroxyl (LOO^.^), and the non-radicals hypochlorous acid (HOCl), ozone (O_3_), singlet oxygen (^1^O_2_), and lipid hydroperoxide (LOOH). When O_2_^.−^ and NO^.^ are produced simultaneously, both radicals react with each other at a diffusion-limited rate to form peroxynitrite (ONOO^−^), which is a good oxidant [1]. Peroxynitrite can induce tyrosine nitration and alter the function of several proteins [2,3]. Moreover, peroxynitrite inactivates several antioxidant enzymes, in particular, mitochondrial SOD by nitration and dityrosine formation [4] and thiol-based antioxidant enzymes by sulfoxidation [5].

### 1.3. Role of ROS in Cardiac Physiology and Homeostasis

Under physiological circumstances, there is an exquisite balance between ROS production and ROS degradation resulting in low steady-state ROS levels. ROS participate in normal cellular function and in cellular homeostasis. They modulate physiological functions by their role in cell signaling and by their effect on gene expression [5]. Redox-sensitive signaling pathways play a key role in many homeostatic and stress-response mechanisms in the heart [6,7]. Versatile redox modifications of key cysteine residues in enzymes are a distinct class of modifications of enzymes. These cysteine-based redox switches in enzymes regulate their activity at the posttranslational level [8].

Reversible oxidation of redox-sensitive cysteine residues also occurs in transcription factors and alters their activities. The transcription factor nuclear factor-erythroid 2 p45-related factor 2 (NRF2) induces the expression of multiple antioxidant genes and cytoprotective genes [9]. The NRF2 system is activated in response to increased H_2_O_2_ levels [10]. NRF2 and its target genes play a critical role in cardiovascular homeostasis via the suppression of oxidative stress. Taken together, redox-sensitive pathways are essential in normal cardiac physiology and homeostasis.

### 1.4. Oxidative Stress

Oxidative stress is the state of a transient or a persistent increase of steady-state ROS levels leading to disturbed signaling pathways and oxidative modification of cellular constituents. Protein oxidation, lipid peroxidation, DNA damage, and oxidative changes of microRNAs may induce cellular dysfunction [5,11]. Myocardial cellular dysfunction secondary to oxidative stress may manifest at the organ level as systolic and diastolic dysfunction [12]. However, cellular dysfunction in the vascular compartment may also lead to vascular/endothelial dysfunction, which impairs coronary perfusion and increases afterload [12]. Oxidative stress may also cause cell death via necrosis or apoptosis [12].

Whereas low-level production of ROS is involved in adaptive processes such as modulation of excitation-contraction coupling, physiological cardiac growth and hypertrophy, and cardiac homeostasis, generation of high levels of ROS and/or more potent oxidants like HO^.^ are involved in the activation of maladaptive processes such as impaired calcium handling, cardiomyocyte hypertrophy, interstitial and perivascular myocardial fibrosis, and apoptosis [6,13]. At the organ level, cardiac hypertrophy is classified as physiological when it is associated with normal cardiac function or as pathological when associated with cardiac dysfunction [14].

**Figure 1:**
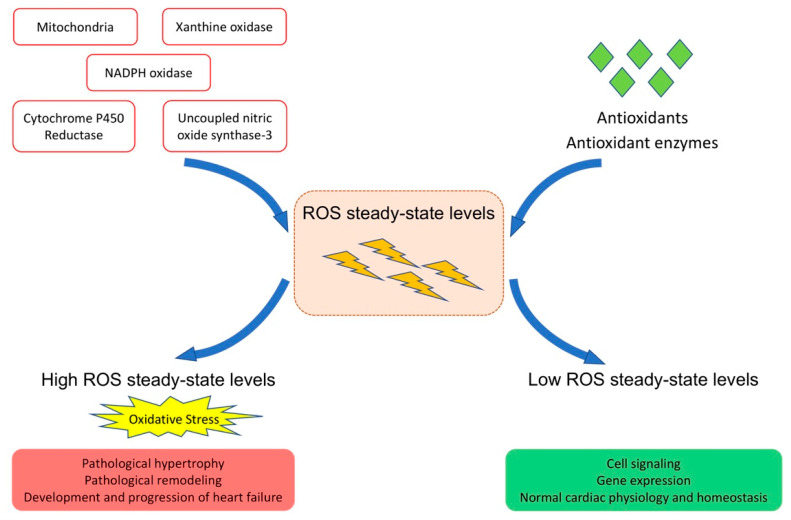
**Role of ROS in cardiac physiology and pathophysiology.** Under physiological conditions, there is an exquisite balance between ROS production and ROS degradation resulting in low steady-state ROS levels. Low levels of ROS have a physiological role in the modulation of excitation-contraction coupling in adaptive processes like physiological cardiac hypertrophy, and in cardiac homeostasis. Oxidative stress resulting from an increase of steady-state ROS levels results in maladaptive processes such as detrimental effects on cardiomyocyte electrophysiology, capillary rarefaction, interstitial and perivascular myocardial fibrosis, and apoptosis. Oxidative stress contributes to pathological hypertrophy, pathological remodeling, and the development and progression of heart failure.

Myocardial fibrosis, apoptosis in the myocardium, and capillary rarefaction are induced by oxidative stress and are features of pathological hypertrophy [13]. Oxidative stress stimulates transforming growth factor-ß1 expression, promotes the transformation of fibroblasts to myofibroblasts, and induces collagen synthesis leading to myocardial fibrosis [15,16]. Independent of structural and ultrastructural alterations, ROS have direct functional effects by affecting proteins central to excitation-contraction coupling, including L-type calcium channels, sodium and potassium channels, and the sodium-calcium exchanger [17]. ROS can alter the activity of sarcoplasmic/endoplasmic reticulum Ca^2+^-ATPase (SERCA) and may decrease myofilament calcium sensitivity [17,18]. All in all, the role of ROS is either physiological or pathological, adaptive or maladaptive, dependent on ROS levels (Figure 1).

### 1.5. Oxidative Stress and Heart Failure

Oxidative stress is a key pathophysiological player in pathological hypertrophy, pathological remodeling, and the development and progression of heart failure. The classical syndrome of heart failure is marked by sodium and water retention leading to pulmonary congestion and peripheral edema (backward failure) and/or a decrease of cardiac output (forward failure). There is direct evidence that H_2_O_2_ and ·OH are generated via O_2_^.-^ within the failing myocardium [19]. Heart failure is often accompanied by myocardial injury as defined by a rise of cardiac troponin levels above the 99th percentile [20,21,22], which generally is due to cardiomyocyte apoptosis or cardiomyocyte necrosis [23].

The heart is the metabolically most active organ and is characterized by the highest content of mitochondria of any tissue. Mitochondria comprise 25–30% of cell volume across different mammalian species [24,25]. Mitochondria are the main source of ROS in the myocardium. Between 0.2% and 2% of electrons may escape the respiratory chain and react with O_2_ leading to the production of O_2_^.−^ during oxidative phosphorylation [5]. Other sources of ROS are NADPH oxidases, xanthine oxidase, cytochrome P450 enzymes, and uncoupling of nitric oxide synthase-3 [5,18,26,27]. The oxidant and nitrating agent peroxynitrite oxidizes tetrahydrobiopterin, an important cofactor of nitric oxide synthase-3. When deprived of its reducing cofactor tetrahydrobiopterin or of its substrate L-arginine, nitric oxide synthase-3 uncouples to the monomeric form that generates O_2_^.−^ rather than nitric oxide, a phenomenon known as uncoupling of nitric oxide synthase-3 [28]. Pressure overload triggers nitric oxide synthase-3 uncoupling as a prominent source of myocardial ROS that contributes to ventricular dilatation and cardiac dysfunction [29].

## 2. Antioxidant Enzyme Gene Transfer

### 2.1. Gene Transfer of the Primary Antioxidant Enzymes

The three primary antioxidant enzymes are superoxide dismutases (SODs), catalase, and glutathione peroxidase. SODs are a family of isoenzymes involved in the scavenging of O_2^−^_ [30]. All mammalian cells possess three isoforms of SOD enzymes: the cytosolic Cu, Zn dimeric form of SOD (SOD1), the mitochondrial tetrameric manganese superoxide dismutase (SOD2), and the extracellular tetrameric Cu, Zn SOD (SOD3). All these enzymes catalyze the same reaction converting O_2_^.−^ in O_2_ and H_2_O_2_ through the alternate reduction and re-oxidation of Cu^2+^ for SOD1 and SOD3 and of Mn^3+^ for SOD2. The enzymes catalase and glutathione peroxidase convert H_2_O_2_ into O_2_ and H_2_O. Enzymatic systems protecting against hydroxyl radicals have never been described and apparently do not exist because of the high reactivity of the hydroxyl radical (HO^.^) [12]. Cysteine disulfides, which constitute an important component in biological redox buffer systems, are highly reactive toward the hydroxyl radical (HO^.^) [31].

There is a relative paucity of studies evaluating the effect of gene transfer of the primary antioxidant enzymes on the myocardium and on heart failure. This is regrettable since gene transfer of the primary antioxidant enzymes is a very suitable tool to specifically evaluate the causal role of oxidative stress in heart failure.

Targeted adeno-associated viral (AAV) serotype 9 gene transfer of SOD3 via the cardiac troponin T-promoter protected against left ventricular remodeling following myocardial infarction in a murine model [32]. The same group had previously demonstrated that a single direct injection into the left ventricular wall of an AAV serotype 9 gene transfer expressing SOD3 under control of the cardiac troponin T-promoter reduced the size of myocardial infarction in mice [33]. Systemic adenoviral gene transfer of SOD3 improved endothelial function in rats with chronic ischemic heart failure [34]. Finally, SOD3 gene transfer improved skeletal muscle abnormalities, cachexia, and exercise intolerance in a murine model of congestive heart failure [35].

Combined gene transfer of adenoviral vectors encoding SOD2 and human catalase via intrapericardial delivery augmented antioxidant enzyme activity and minimized contractile dysfunction after ischemic reperfusion in the isolated perfused neonatal mouse heart [36].

Overexpression of glutathione peroxidase in transgenic mice attenuated left ventricular remodeling and development of heart failure after myocardial infarction in mice [13]. Moreover, overexpression of glutathione peroxidase resulted in an improvement of left ventricular diastolic function, attenuation of cardiomyocyte hypertrophy, a decrease of interstitial fibrosis, and a reduction of apoptosis in a model of streptozotocin-induced diabetes mellitus [37]. Conversely, glutathione peroxidase-1 deficiency accelerated cardiac hypertrophy and dysfunction in a model of angiotensin II-induced hypertension [38]. Unfortunately, gene transfer intervention studies of glutathione peroxidase have not been performed in experimental models of heart failure.

### 2.2. Thioredoxin Gene Transfer

Thioredoxin is a small 12-kDa redox-acting protein consisting of 105 amino acids that are ubiquitously present in the human body [39]. The thioredoxin system comprises thioredoxin, thioredoxin reductase, and NADPH. Thioredoxin reductase and NADPH maintain the reducing activity of thioredoxin. In mammals, there are at least three members in the thioredoxin family [40]. In the broad sense, the thioredoxin system also includes thioredoxin peroxidase (peroxiredoxin) [41]. Thioredoxin reduces peroxiredoxin, which then catalyzes the conversion of H_2_O_2_ to H_2_O.

Thioredoxin exchanges disulfide to dithiol to maintain the reducing status of various intracellular molecules. The active-site CXXC motif of this thiol: disulfide oxidoreductase is essential for its catalysis of redox reactions. In the cytoplasm, thioredoxin acts as a radical scavenger, either by itself or in cooperation with peroxiredoxin [39]. Human thioredoxin is a powerful singlet oxygen quencher and hydroxyl radical (HO^.^) scavenger [42,43]. Thioredoxin is a multifunctional protein and has not only antioxidative effects but also anti-inflammatory and antiapoptotic effects [39].

Intramyocardial gene transfer with an adenoviral vector encoding thioredoxin-1 immediately after myocardial infarction in diabetic rats reduced oxidative stress in the myocardium, decreased myocardial fibrosis, reduced cardiomyocyte and endothelial cell apoptosis, increased capillary and arteriolar density in the myocardium, and resulted in the preservation of myocardial function compared to control vector-treated rats [44]. These preclinical findings strongly suggest that a precisely balanced antioxidant system is essential for the maintenance of cardiac function in the setting of diabetes.

### 2.3. Heme Oxygenase-1 Gene Transfer

**Figure 2:**
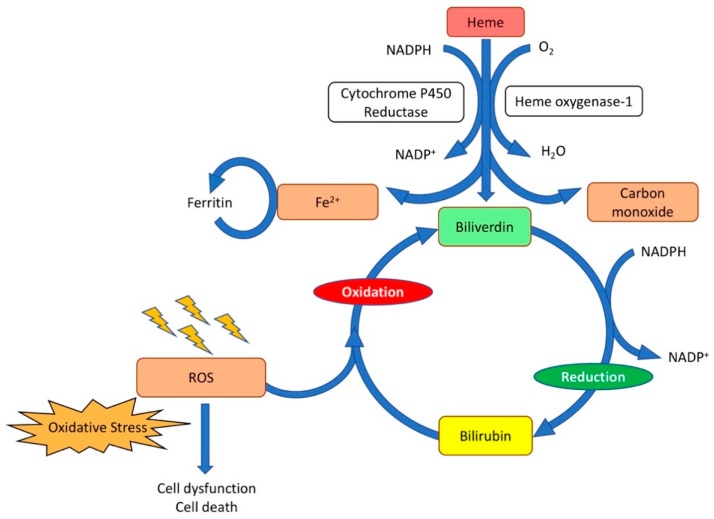
**Catabolism of heme.** Heme oxygenase is the rate-limiting enzyme in the catabolism of heme. In connection with cytochrome P450 reductase and in the presence of NADPH and three molecules of molecular oxygen (O_2_) per heme molecule, it catalyzes the oxidative cleavage of heme to render equimolar amounts of biliverdin, ferrous iron (Fe^2+^), and carbon monoxide. Ferrous iron (Fe^2+^) is sequestered into ferritin for storage. In the second reaction, the green pigment biliverdin is reduced to the yellow pigment bilirubin by biliverdin reductase. Bilirubin can serve as an antioxidant and reverts back to biliverdin. The biliverdin–bilirubin–biliverdin cycle is very powerful to detoxify ROS.

Another enzyme with an antioxidative potential is heme oxygenase-1. This enzyme is an inducible stress response protein that exerts pleiotropic cytoprotective effects, including reduction of oxidative stress [45,46,47], inflammation [48], and apoptosis [49]. Heme can be derived either from intracellular sources, such as hemoproteins and mitochondria, or from extracellular sources, namely damaged tissues, and red blood cell hemolysis. Heme oxygenase-1 converts heme to biliverdin [50]. This reaction also generates carbon monoxide (CO) and induces the release of iron, which is stored within the iron-binding protein ferritin [45,51]. Biliverdin is subsequently reduced to bilirubin by biliverdin reductase (Figure 2). Bilirubin can scavenge ROS and is reconverted to biliverdin [50].

The global role of heme oxygenase-1 in the heart is complex. This enzyme is a master protective sentinel [52]. Tissue damage results in the release of heme, and heme upregulates heme oxygenase-1 [52]. Carbon monoxide produced by heme oxygenase-1 has multiple effects on mitochondria. It modulates the enzymatic activity of cytochrome c oxidase, results in the generation of ROS for signaling by mitochondrial oxidases, and induces a mild mitochondrial uncoupling effect [53]. The carbon monoxide-induced increase of ROS results in signaling leading to the expression of antioxidant genes (e.g., SOD2, thioredoxins) as well as heme oxygenase-1 and the transcription factor NRF2 [52]. The powerful antioxidant bilirubin generated from biliverdin by biliverdin reductase serves to ultimately resolve the oxidative burden [52].

Intramyocardial injection of AAV vectors encoding human heme oxygenase-1 resulted in a reduction of infarct size in a rat model of ischemia-reperfusion injury [54]. The reduction in infarct size was accompanied by reductions in myocardial lipid peroxidation, in proapoptotic BAX, also known as BCL-2-like protein 4, and in the proinflammatory interleukin-1β protein abundance, concomitant with an increase in antiapoptotic BCL-2 protein level [54]. In a porcine model of ischemia-reperfusion injury, retrograde infusion of AAV human heme oxygenase-1 vectors in the anterior ventricular vein resulted in smaller infarct size, better preservation of ejection fraction, and in a markedly reduced post-ischemic influx of myeloperoxidase-positive neutrophils and CD14(+) monocytes compared to control animals [55].

Intramyocardial injection of AAV serotype 2 vectors encoding human heme oxygenase-1 has also been investigated in a rat model of cardiac remodeling and development of chronic heart failure [56]. Acute myocardial ischemia/reperfusion injury was induced six weeks after gene transfer by ligation of the proximal left anterior descending coronary artery for 30 min, followed by reperfusion. The heme oxygenase-1-treated animals were characterized by preservation of left ventricular function and left ventricular dimensions and structure one year after myocardial infarction, whereas the control vector-treated rats showed impaired left ventricular function, left ventricular dilatation, and overt signs of heart failure [56].

These results are further corroborated by investigations in transgenic animals. Transgenic mice with cardiomyocyte-specific overexpression of heme oxygenase-1 under control of *α-myosin heavy chain* promoter exhibited significantly improved cardiac ejection fraction and survival following ligation of the left anterior descending coronary artery [57]. Furthermore, cardiac hypertrophy, interstitial fibrosis, and oxidative stress were reduced in this model of chronic ischemic heart failure [57]. Moreover, long-term induction of heme oxygenase-1 by chronic hemin administration exerted protective effects in a rat model of chronic heart failure induced by permanent ligation of the left anterior descending coronary artery [58]. The ischemic hearts of the hemin-treated Sprague-Dawley rats showed a reduction of oxidative stress and a decrease of apoptosis compared to non-treated animals, as evidenced by the decreased levels of lipid peroxidation, free-radical-induced DNA damage, caspase-3 activity, and BAX expression [58].

## 3. Metabolic Risk Factors, Oxidative Stress, and Heart Failure: Impact of Gene Therapy

The thiobarbituric acid reactive substances (TBARS) assay is widely used as a generic metric of lipid peroxidation in biological samples. Plasma TBARS expressed as plasma malondialdehyde equivalents, constitute a good indicator of the levels of systemic oxidative stress. Several metabolic risk factors are associated with prominent systemic oxidative stress [59,60,61,62] and are also linked with an increased risk of heart failure. Multiple gene transfer prevention and intervention studies have demonstrated that cardiac function is improved and heart failure prevented or reversed following gene therapy directed at correction of these metabolic risk factors [63,64]. Gene therapy directed at correction of metabolic risk factors also resulted in a marked reduction of systemic oxidative stress and of oxidative stress in the myocardium [63,64]. These studies do not prove an obligatory cause-and-effect relationship between the decrease of oxidative stress and cardiac phenotype. However, the consistency of the data suggests that reduction of oxidative stress in these studies is an important mediator of the observed effects of metabolic gene therapy on cardiac structure and function.

Homocysteine contains a highly reactive thiol group that can undergo disulfide exchange reactions with cysteine residues in different proteins [65] or results in auto-oxidation and the formation of ROS [66]. Selective homocysteine-lowering gene transfer with an E1E3E4-deleted adenoviral vector AdCBS, which induces hepatocyte-specific expression of cystathionine-β-synthase (CBS), in a murine model of hyperhomocysteinemia, attenuated pressure overload-induced cardiomyopathy via reduced oxidative stress [67]. Gene therapy in this model strikingly reduced plasma TBARS levels and the myocardial 3-nitrotyrosine-positive area (%) following transverse aortic constriction. Left ventricular hypertrophy, apoptosis in the myocardium, and interstitial myocardial fibrosis induced by pressure overload were markedly lower following AdCBS gene transfer. Homocysteine-lowering gene transfer significantly improved diastolic function in mice with pressure overload and congestive heart failure was reduced by pre-emptive AdCBS gene transfer as evidenced by the decrease of the wet lung weight [67]. Based on a similar gene therapy approach, beneficial effects of selective homocysteine-lowering gene therapy were also manifest in a model of chronic ischemic heart failure induced by permanent ligation of the left anterior descending coronary artery [68]. Taken together, these studies strongly suggest that reduction of oxidative stress contributes to inhibition of the pathological remodeling of the heart.

Hypercholesterolemia is associated with oxidative stress [59,69,70]. Mitochondria from hypercholesterolemic low-density lipoprotein (LDL) receptor (LDLr)-deficient mice have preserved oxidative phosphorylation efficiency but a higher net production of ROS [71]. AAV serotype 8 (AAV8)-mediated low-density lipoprotein receptor (LDLr) (AAV8-LDLr) gene transfer potently reduced plasma cholesterol levels in C57BL/6 LDLr^−/−^ mice [72,73]. AAV8-LDLr gene transfer attenuated left ventricular hypertrophy induced by transverse aortic constriction. Moreover, interstitial myocardial fibrosis and perivascular myocardial fibrosis were significantly reduced in mice with pressure overload following AAV8-LDLr gene transfer. Cholesterol-lowering gene therapy also improved systolic and diastolic cardiac function after transverse aortic constriction and counteracted heart failure, as indicated by the pronounced reduction of wet lung weight. AAV8-LDLr gene transfer decreased the myocardial 3-nitrotyrosine-positive area and systemic TBARS levels. Taken together, reduction of oxidative stress may be an important mediator of the observed favorable effects of cholesterol-lowering gene therapy in models of pressure overload-induced cardiomyopathy [72,73]. Similar results have been demonstrated in a model of chronic ischemic heart failure [74].

Obesity, the metabolic syndrome, and diabetes mellitus are characterized by chronic low-grade inflammation with permanently increased oxidative stress [61,62,75]. In a murine model of diabetic cardiomyopathy induced by feeding a high-sugar/high-fat diet in LDLr^−/−^ mice, cholesterol-lowering AAV8-LDLr gene therapy potently reduced oxidative stress and plasma tumor necrosis factor-α levels [76]. AAV8-LDLr gene transfer prominently counteracted pathological remodeling and preserved cardiac function in mice fed this high-sugar/high-fat diet [76].

High-density lipoproteins (HDL) have a strong antioxidative potential [77,78,79,80]. Apolipoprotein (apo) A-I is the main apolipoprotein of HDL. Gene transfer with an E1E3E4-deleted human *apo A-I* gene transfer vector, which selectively increases HDL, reduces oxidative stress, inflammation, and myocardial fibrosis in a rat model of diabetic cardiomyopathy [81]. Increased HDL following human *apo A-I* gene transfer has also been shown to prevent endothelial nitric oxide synthase uncoupling in diabetes mellitus, which may contribute to a decrease in ROS production [82,83]. Moreover, selective HDL-raising AAV serotype 8-human *apo A-I* (AAV8-A-I) gene transfer resulted in a potent reduction of oxidative stress and prevented pathological remodeling following transverse aortic constriction in mice [84]. The HDL receptor scavenger receptor class B, type I (SR-BI), mediates the selective uptake of HDL lipids, including lipid hydroperoxides [85,86]. *Scarb1*-deficient mice, lacking SR-BI protein expression, are characterized by increased plasma cholesterol comprising predominantly enlarged HDL enriched in free cholesterol and apolipoprotein E [87] and by HDL dysfunction including a reduced antioxidative potential resulting in increased oxidative stress [86,88]. Dysfunctional HDL in *Scarb1^−/−^* mice augmented oxidative stress, potentiated cardiac hypertrophy induced by transverse aortic constriction, and aggravated pressure overload-induced cardiomyopathy [89]. Increased oxidative stress in *Scarb1^−/−^* mice was rescued by gene transfer with an E1E3E4-deleted adenoviral vector containing a hepatocyte-specific SR-BI-encoding expression cassette [89,90]. Gene transfer with this vector also potently counteracted pathological remodeling and development of heart failure in mice subjected to transverse aortic constriction. 

All in all, gene therapy prevention and intervention studies directed at correcting metabolic risk factors are broadly robust in demonstrating a plausible link between reduced oxidative stress and prevention or reversal of heart failure.

## 4. Gene Transfer of MicroRNAs and Modulation of MicroRNA Activity to Reduce Oxidative Stress and to Prevent Heart Failure

### 4.1. MicroRNAs and Oxidative Stress

MicroRNAs are natural, endogenous and single-stranded molecules consisting of approximately 22 non-coding nucleotides and constitute a very important family of gene regulators [91]. They derive from longer RNA transcripts that frequently originate from genomic sequences embedded in introns. MicroRNAs bind to complementary target sequences on the 3′ untranslated region of specific mRNAs and regulate their expression. This silencing effect occurs by either repressing translation of the transcript or promoting the degradation of the transcript. A recent estimation of the total number of microRNAs in the human genome yielded a value of 2300, but this is an extrapolated value based on a number of assumptions [92]. MicroRNAs are estimated to affect up to 60% of protein-coding genes [93].

Oxidative stress may alter the expression levels of many microRNAs in cardiovascular disease and heart failure [94,95]. Redox-sensitive microRNAs constitute potential therapeutic targets for oxidative-stress-related heart diseases and heart failure. Manipulation of gene expression using microRNA gene transfer, microRNA mimics, or microRNA antagomirs can at least theoretically be applied to reduce oxidative stress in heart failure. MicroRNA mimics are applied to induce gene silencing of genes that directly or indirectly increase oxidative stress. MicroRNA mimic technology is an approach for gene silencing based on the introduction of artificial, non-natural double-stranded microRNA-like RNA fragments [96]. Unlike endogenous microRNAs, microRNA mimics act in a gene-specific fashion [96]. In contrast, microRNA antagomirs are chemically designed oligonucleotides that specifically inhibit target microRNA molecules by complementary binding to them, which results in an upregulation of genes.

Alternatively, microRNA sponges are applied to induce microRNA loss-of-function. These sponges contain multiple tandem complementary microRNA antisense binding sites for the microRNA of interest and therefore sequester microRNAs from their endogenous targets [97,98]. Viral gene therapy vectors may be an adequate tool for microRNA sponge-based therapy by inducing a high level of the sponge and by inducing prolonged expression of the sponge [99]. Limitations of these strategies are that a single microRNA may target many different genes limiting the specificity of these interventions. Secondly, individual mRNAs are targeted by multiple microRNAs, which may attenuate the effect of targeting one single microRNA.

Since the field of microRNAs, oxidative stress, and gene transfer is very broad, the discussion in the next paragraphs is restricted to key studies and concepts.

### 4.2. Impact of Oxidative Changes of MicroRNAs on Cardiac Hypertrophy and Heart Failure: Direct Evidence for a Causal Role of Oxidative Stress

The oxidation of guanine within nucleic acids produces 8-oxoguanine (o^8^G), which can pair with adenine and induce guanine-to-thymine (G > T) mutations in DNA. RNA is more vulnerable than DNA to this modification [100]. MicroRNAs may be oxidatively modified by ROS, leading to misrecognition of target mRNAs. ROS-induced oxidized guanine (o^8^G) appears to play a key role in this respect. ROS could induce a shift in the pool of target microRNAs, depending on the oxidation state of the microRNAs [11]. Cardiac hypertrophy is a major contributor to the development of heart failure and has long been associated with oxidative stress [101]. In in vitro and in vivo models of hypertrophic stimulation by adrenergic receptor agonists, position-specific o^8^G modifications were generated in seed regions (positions 2–8) of selective microRNAs, which resulted in the regulation of other mRNAs through o^8^G•A base pairing [11]. The authors demonstrated that o^8^G is induced predominantly at position 7 of miR-1 (7o^8^G-miR-1) by treatment with adrenergic agonists. The pathogenic importance of this oxidation of guanine is underscored by experiments showing that introducing 7o^8^G-miR-1 or 7U-miR-1 (in which G at position 7 is substituted with U) alone is sufficient to cause cardiac hypertrophy in mice. Furthermore, 7o^8^G-miR-1 globally redirects target repression [11]. Causality was further demonstrated by experiments showing that the specific inhibition of 7o^8^G-miR-1 in mouse cardiomyocytes by use of microRNA sponges was found to attenuate hypertrophy [11]. Finally, the authors demonstrated that o^8^G-miR-1 is also implicated in patients with cardiomyopathy [11]. Taken together, these experimental approaches underscore that oxidative changes of microRNAs play a causal role in cardiac hypertrophy.

### 4.3. MiR-152 Gene Transfer to Reduce Oxidative Stress and to Improve Cardiac Function in a Model of Doxorubicin-Induced Cardiomyopathy

Doxorubicin is the most frequently prescribed anticancer chemotherapeutic and is administered as a single agent or in combination with other antitumor drugs [102]. Administration of doxorubicin can induce both short- and long-term cardiotoxic effects, which range from subclinical alterations of myocardial structure and function to severe cardiomyopathy and heart failure [102]. Oxidative stress plays a major role in doxorubicin-induced cardiotoxicity [102,103].

As already stated in Section 1, NRF2 is a transcription factor that regulates the expression of antioxidant proteins [9]. NRF2 binds to the antioxidant responsive element (ARE) [104]. Activation of this pathway protects cells from oxidative stress-induced cell death [104]. Under physiological conditions, NRF2 binds Kelch-like ECH-associated protein 1 (KEAP1) and the Cullin 3 (CUL3)-based E3 ubiquitin ligase [105,106]. Following stimulation, NRF2 is released from KEAP1 and can bind to the ARE, which results in the transcription of NRF2-dependent antioxidant genes [105,106].

Downregulation of miR-152 was observed following doxorubicin treatment in mice [105]. AAV serotype 9 gene transfer of miR-152 under control of the *cardiac troponin T* promoter protected doxorubicin-treated mice against oxidative stress and resulted in an attenuation of doxorubicin-induced cardiac injury and an improvement of cardiac function [105]. Cardiac protection was conferred via activation of NRF2. MiR-152 targets the 3′-UTR of KEAP1 and decreases KEAP1 levels. Increased NRF2 activity as a result of decreased KEAP 1 levels increased expression of the NRF2-dependent genes heme oxygenase-1, NAD(P)H quinone dehydrogenase 1, and SOD. NRF2 activation appeared to be essential for miR-152-induced cardioprotection [105].

### 4.4. MicroRNA-132 Inhibition Using Antagomirs

MiR-132 is a prognostic biomarker in heart failure patients [107] and has a significant impact on oxidative stress in the myocardium. Sirtuin 1 expression is a direct target of miR-132 [108,109]. The sirtuins are a family of nicotinamide adenine dinucleotide (NAD)-dependent histone deacetylases (HDACs) that play key roles in histone deacetylation and protein deacetylation [110,111]. The sirtuin family of seven enzymes is involved in cellular antioxidant and redox signaling pathways. The sirtuins promote antioxidant defense and reduce oxidative stress-related processes [112]. In this regard, their deacetylase activity is dependent on NAD^+^, a key redox signaling molecule. Sirtuin 1 significantly enhances the activity of the KEAP1/NRF2/ARE pathway by decreasing KEAP1 expression [113]. Sirtuin 1 induces deacetylation of peroxisome proliferator-activated receptor-γ coactivator (PGC)-1α and stimulates PGC-1α transcriptional activity [114]. PGC-1α is a master regulator of mitochondrial biogenesis and function, including oxidative phosphorylation and ROS detoxification [115]. MiR-132 also directly targets the anti-hypertrophic and pro-autophagic Forkhead box O3 (FOXO3) transcription factor [116]. FOXO3 also protects cells from oxidative stress [117,118]. Since microRNA-132 negatively regulates the expression of Sirtuin 1, pharmacological inhibition of miR-132 by an antagomir increases Sirtuin1 and activates PGC-1α and NRF2 signaling.

Pharmacological inhibition of miR-132 also enhances FoxO3 activity. These effects lead to an inhibition of oxidative stress [119,120]. Antisense therapy targeting miR-132 has been evaluated in several preclinical models of heart failure [121,122]. CDR132L, a specific antisense oligonucleotide targeting miR-132, has also been evaluated in phase 1b, randomized, double-blind, placebo-controlled study in humans [123]. CDR132L was safe and well-tolerated. Treatment induced significant narrowing of the QRS complex. Moreover, preliminary data suggest positive effects on relevant cardiac fibrosis biomarkers [123]. Reduction of oxidative stress is likely an important mediator of the effect of CDR132L. Nevertheless, it should be kept in mind that miR-132 also targets other important genes involved in cardiac function and remodeling. MiR-132 downregulates the expression of genes involved in intracellular calcium handling and contractility, e.g., SERCA2A [96].

## 5. Conclusions

Gene transfer studies have provided strong corroborative evidence for a key role of oxidative stress in pathological cardiac hypertrophy and remodeling and in the development of heart failure. Since the heart is the metabolically most active organ and is characterized by the highest content of mitochondria of any tissue, this organ is very susceptible and vulnerable to oxidative stress. Oxidative stress not only causes protein oxidation, lipid peroxidation, and DNA damage but also oxidative changes of microRNAs [5,11]. The key pathogenetic role of oxidative changes of microRNAs has been unequivocally demonstrated [11].

Furthermore, the cellular levels of redox-sensitive microRNAs are altered in response to oxidative stress, and increasing evidence indicates that these redox-sensitive microRNAs constitute potential therapeutic targets for the treatment of heart failure [105,121,122,123]. The causal role of oxidative stress in heart failure is also supported by gene transfer studies of the three primary antioxidant enzymes (SODs, catalase, and glutathione peroxidase), of thioredoxin, and of heme oxygenase-1. Finally, multiple gene transfer prevention and intervention studies have demonstrated that gene therapy directed at the correction of metabolic risk factors resulted in a marked reduction of systemic oxidative stress and oxidative stress in the myocardium [63,64]. This correction of metabolic risk factors also resulted in improved cardiac function and prevention or reversal of pathological remodeling and heart failure [63,64]. The broad robustness of the strong link between reduction of oxidative stress and prevention and treatment of heart failure suggests that reduction of oxidative stress is an important mediator of the observed effects of metabolic gene therapy on cardiac structure and function.

Heart failure is the cardiovascular epidemic of this century and has a rather dismal prognosis [124]. The development of gene transfer strategies that result in an improved cellular redox state remains an important research area in the generation of new treatments for heart failure.

## References

[1] Radi R. (2013). Peroxynitrite, a Stealthy Biological Oxidant. J. Biol. Chem..

[2] Zou M.-H., Bachschmid M.M. (1999). Hypoxia–Reoxygenation Triggers Coronary Vasospasm in Isolated Bovine Coronary Arteries via Tyrosine Nitration of Prostacyclin Synthase. J. Exp. Med..

[3] Weber M., Lauer N., Mülsch A., Kojda G. (2001). The effect of peroxynitrite on the catalytic activity of soluble guanylyl cyclase. Free Radic. Biol. Med..

[4] MacMillan-Crow L.A., Crow J.P., Thompson J.A. (1998). Peroxynitrite-mediated inactivation of manganese superoxide dis-mutase involves nitration and oxidation of critical tyrosine residues. Biochemistry.

[5] Münzel T., Gori T., Keaney J.F., Maack C., Daiber A. (2015). Pathophysiological role of oxidative stress in systolic and diastolic heart failure and its therapeutic implica-tions. Eur. Heart J..

[6] Santos C.X., Anilkumar N., Zhang M., Brewer A.C., Shah A.M. (2011). Redox signaling in cardiac myocytes. Free Radic. Biol. Med..

[7] Rhee S.G. (1999). Redox signaling: Hydrogen peroxide as intracellular messenger. Exp. Mol. Med..

[8] Klomsiri C., Karplus P., Poole L.B. (2011). Cysteine-Based Redox Switches in Enzymes. Antioxid. Redox Signal..

[9] Zhou S., Sun W., Zhang Z., Zheng Y. (2014). The Role of Nrf2-Mediated Pathway in Cardiac Remodeling and Heart Failure. Oxidative Med. Cell. Longev..

[10] Fourquet S., Guerois R., Biard D., Toledano M.B. (2010). Activation of NRF2 by Nitrosative Agents and H2O2 Involves KEAP1 Disulfide Formation. J. Biol. Chem..

[11] Seok H., Lee H., Lee S., Ahn S.H., Lee H.-S., Kim G.-W.D., Peak J., Park J., Cho Y.K., Jeong Y. (2020). Position-specific oxidation of miR-1 encodes cardiac hypertrophy. Nat. Cell Biol..

[12] Lushchak V.I. (2015). Free radicals, reactive oxygen species, oxidative stresses and their classifications. Ukr. Biochem. J..

[13] Shiomi T., Tsutsui H., Matsusaka H., Murakami K., Hayashidani S., Ikeuchi M., Wen J., Kubota T., Utsumi H., Takeshita A. (2004). Overexpression of Glutathione Peroxidase Prevents Left Ventricular Remodeling and Failure After Myocardial Infarction in Mice. Circulation.

[14] Shimizu I., Minamino T. (2016). Physiological and pathological cardiac hypertrophy. J. Mol. Cell. Cardiol..

[15] Zhao W., Zhao T., Chen Y., Ahokas R.A., Sun Y. (2008). Oxidative stress mediates cardiac fibrosis by enhancing transforming growth factor-beta1 in hypertensive rats. Mol. Cell. Biochem..

[16] Philip J.L., Razzaque M.A., Han M., Li J., Theccanat T., Xu X., Akhter S.A. (2015). Regulation of mitochondrial oxidative stress by beta-arrestins in cultured human cardiac fibroblasts. Dis. Model Mech..

[17] Takimoto E., Kass D.A. (2007). Role of Oxidative Stress in Cardiac Hypertrophy and Remodeling. Hypertens.

[18] van der Pol A., van Gilst W.H., Voors A.A., van der Meer P. (2019). Treating oxidative stress in heart failure: Past, present and future. Eur. J. Heart Fail..

[19] Ide T., Tsutsui H., Kinugawa S., Suematsu N., Hayashidani S., Ichikawa K., Utsumi H., Machida Y., Egashira K., Takeshita A. (2000). Direct Evidence for Increased Hydroxyl Radicals Originating from Superoxide in the Failing Myocardium. Circ. Res..

[20] Thygesen K., Alpert J.S., Jaffe A.S., Chaitman B.R., Bax J.J., Morrow D.A., White H.D. (2018). Fourth Universal Definition of Myocardial Infarction (2018). J. Am. Coll. Cardiol..

[21] Sato Y., Fujiwara H., Takatsu Y. (2012). Cardiac troponin and heart failure in the era of high-sensitivity assays. J. Cardiol..

[22] Kociol R., Pang P., Gheorghiade M., Fonarow G., O’Connor C.M., Felker G. (2010). Troponin Elevation in Heart Failure: Prevalence, Mechanisms, and Clinical Implications. J. Am. Coll. Cardiol..

[23] Mair J., Lindahl B., Hammarsten O., Müller C., Giannitsis E., Huber K., Möckel M., Plebani M., Thygesen K., Jaffe A.S. (2018). How is cardiac troponin released from injured myocardium?. Eur. Heart J. Acute Cardiovasc. Care.

[24] Barth E. (1992). Ultrastructural quantitation of mitochondria and myofilaments in cardiac muscle from 10 different animal species including man. J. Mol. Cell. Cardiol..

[25] Schaper J., Meiser E., Stämmler G. (1985). Ultrastructural morphometric analysis of myocardium from dogs, rats, hamsters, mice, and from human hearts. Circ. Res..

[26] Lu D., Ma Y., Zhang W., Bao D., Dong W., Lian H., Huang L., Zhang L. (2012). Knockdown of cytochrome P450 2E1 inhibits oxidative stress and apoptosis in the cTnT(R141W) dilated cardi-omyopathy transgenic mice. Hypertension.

[27] Zhao G.-J., Zhao C.-L., Ouyang S., Deng K.-Q., Zhu L., Montezano A.C., Zhang C., Hu F., Zhu X.-Y., Tian S. (2020). Ca^2+^ -Dependent NOX5 (NADPH Oxidase 5) Exaggerates Cardiac Hypertrophy Through Reactive Oxygen Species Production. Hypertension.

[28] Forstermann U., Munzel T. (2006). Endothelial nitric oxide synthase in vascular disease: From marvel to menace. Circulation.

[29] Takimoto E., Champion H.C., Li M., Ren S., Rodriguez E.R., Tavazzi B., Lazzarino G., Paolocci N., Gabrielson K.L., Wang Y. (2005). Oxidant stress from nitric oxide synthase–3 uncoupling stimulates cardiac pathologic remodeling from chronic pressure load. J. Clin. Investig..

[30] Mondola P., Damiano S., Sasso A., Santillo M. (2016). The Cu, Zn Superoxide Dismutase: Not Only a Dismutase Enzyme. Front. Physiol..

[31] Adhikari S., Crehuet R., Anglada J.M., Francisco J.S., Xia Y. (2020). Two-step reaction mechanism reveals new antioxidant capability of cysteine disulfides against hydroxyl radical attack. Proc. Natl. Acad. Sci. USA.

[32] Konkalmatt P.R., Beyers R.J., O’Connor D.M., Xu Y., Seaman M.E., French B.A. (2013). Cardiac-Selective Expression of Extracellular Superoxide Dismutase After Systemic Injection of Adeno-Associated Virus 9 Protects the Heart Against Post–Myocardial Infarction Left Ventricular Remodeling. Circ. Cardiovasc. Imaging.

[33] Prasad K.M.R., Smith R.S., Xu Y., French B.A. (2011). A single direct injection into the left ventricular wall of an adeno-associated virus 9 (AAV9) vector ex-pressing extracellular superoxide dismutase from the cardiac troponin-T promoter protects mice against myocardial infarction. J. Gene Med..

[34] Iida S., Chu Y., Francis J., Weiss R.M., Gunnett C.A., Faraci F., Heistad D.D. (2005). Gene transfer of extracellular superoxide dismutase improves endothelial function in rats with heart failure. Am. J. Physiol. Circ. Physiol..

[35] Okutsu M., Call J.A., Lira V.A., Zhang M., Donet J.A., French B.A., Martin K.S., Peirce S., Rembold C.M., Annex B.H. (2014). Extracellular Superoxide Dismutase Ameliorates Skeletal Muscle Abnormalities, Cachexia, and Exercise Intolerance in Mice with Congestive Heart Failure. Circ. Heart Fail..

[36] Woo Y.J., Zhang J.C., Vijayasarathy C., Zwacka R., Englehardt J.F., Gardner T.J., Sweeney H.L. (1998). Recombinant adenovirus-mediated cardiac gene transfer of superoxide dismutase and catalase attenuates postischemic contractile dysfunction. Circulation.

[37] Matsushima S., Kinugawa S., Ide T., Matsusaka H., Inoue N., Ohta Y., Yokota T., Sunagawa K., Tsutsui H. (2006). Overexpression of glutathione peroxidase attenuates myocardial remodeling and preserves diastolic function in diabetic heart. Am. J. Physiol. Circ. Physiol..

[38] Ardanaz N., Yang X.-P., Cifuentes M.E., Haurani M.J., Jackson K.W., Liao T.-D., Carretero O.A., Pagano P.J. (2010). Lack of Glutathione Peroxidase 1 Accelerates Cardiac-Specific Hypertrophy and Dysfunction in Angiotensin II Hypertension. Hypertension.

[39] Hoshino Y., Shioji K., Nakamura H., Masutani H., Yodoi J. (2007). From Oxygen Sensing to Heart Failure: Role of Thioredoxin. Antioxid. Redox Signal..

[40] Watson W.H., Yang X., Choi Y.E., Jones D.P., Kehrer J.P. (2004). Thioredoxin and Its Role in Toxicology. Toxicol. Sci..

[41] Ago T., Sadoshima J. (2006). Thioredoxin and ventricular remodeling. J. Mol. Cell. Cardiol..

[42] Das K.C., Das C.K. (2000). Thioredoxin, a singlet oxygen quencher and hydroxyl radical scavenger: Redox independent func-tions. Biochem. Biophys. Res. Commun..

[43] Shioji K., Kishimoto C., Nakamura H., Masutani H., Yuan Z., Oka S.-I., Yodoi J. (2002). Overexpression of Thioredoxin-1 in Transgenic Mice Attenuates Adriamycin-Induced Cardiotoxicity. Circulation.

[44] Samuel S.M., Thirunavukkarasu M., Penumathsa S.V., Koneru S., Zhan L., Maulik G., Sudhakaran P.R., Maulik N. (2010). Thioredoxin-1 gene therapy enhances angiogenic signaling and reduces ventricular remodeling in in-farcted myocardium of diabetic rats. Circulation.

[45] Balla G., Jacob H.S., Balla J., Rosenberg M., Nath K., Apple F., Eaton J.W., Vercellotti G.M. (1992). Ferritin: A cytoprotective antioxidant strategem of endothelium. J. Biol. Chem..

[46] Poss K.D., Tonegawa S. (1997). Reduced stress defense in heme oxygenase 1-deficient cells. Proc. Natl. Acad. Sci. USA.

[47] Stocker R., Yamamoto Y., McDonagh A.F., Glazer A.N., Ames B.N. (1987). Bilirubin Is an Antioxidant of Possible Physiological Importance. Science.

[48] Wagener F.A.D.T.G., Volk H.-D., Willis D., Abraham N.G., Soares M., Adema G.J., Figdor C. (2003). Different Faces of the Heme-Heme Oxygenase System in Inflammation. Pharmacol. Rev..

[49] Katori M., Buelow R., Ke B., Ma J., Coito A.J., Iyer S., Southard D., Busuttil R.W., Kupiec-Weglinski J.W. (2002). Heme Oxygenase-1 Overexpression Protects Rat Hearts from Cold Ischemia/Reperfusion Injury Via an Antiapoptotic Pathway. Transplantation.

[50] Sedlak T.W., Snyder S.H. (2006). Messenger molecules and cell death: Therapeutic implications. JAMA.

[51] Balla J., Jacob H.S., Balla G., Nath K., Eaton J.W., Vercellotti G.M. (1993). Endothelial-cell heme uptake from heme proteins: Induction of sensitization and desensitization to oxidant damage. Proc. Natl. Acad. Sci. USA.

[52] Otterbein L.E., Foresti R., Motterlini R. (2016). Heme Oxygenase-1 and Carbon Monoxide in the Heart: The Balancing Act Be-tween Danger Signaling and Pro-Survival. Circ. Res..

[53] Almeida A.S., Figueiredo-Pereira C., Vieira H.L. (2015). Carbon monoxide and mitochondria-modulation of cell metabolism, redox response and cell death. Front. Physiol..

[54] Melo L.G., Agrawal R., Zhang L., Rezvani M., Mangi A.A., Ehsan A., Griese D.P., Dell’Acqua G., Mann M.J., Oyama J. (2002). Gene therapy strategy for long-term myocardial protection using adeno-associated virus-mediated delivery of heme oxygenase gene. Circulation.

[55] Hinkel R., Lange P., Petersen B., Gottlieb E., Ng J.K.M., Finger S., Horstkotte J., Lee S., Thormann M., Knorr M. (2015). Heme Oxygenase-1 Gene Therapy Provides Cardioprotection Via Control of Post-Ischemic Inflammation: An Experimental Study in a Pre-Clinical Pig Model. J. Am. Coll. Cardiol..

[56] Liu X., Simpson J.A., Brunt K.R., Ward C.A., Hall S.R.R., Kinobe R.T., Barrette V., Tse M.Y., Pang S.C., Pachori A.S. (2007). Preemptive heme oxygenase-1 gene delivery reveals reduced mortality and preservation of left ventricular function 1 yr after acute myocardial infarction. Am. J. Physiol. Circ. Physiol..

[57] Wang G., Hamid T., Keith R.J., Zhou G., Partridge C.R., Xiang X., Kingery J.R., Lewis R.K., Li Q., Rokosh G. (2010). Cardioprotective and Antiapoptotic Effects of Heme Oxygenase-1 in the Failing Heart. Circulation.

[58] Collino M., Pini A., Mugelli N., Mastroianni R., Bani D., Fantozzi R., Papucci L., Fazi M., Masini E. (2013). Beneficial effect of prolonged heme oxygenase 1 activation in a rat model of chronic heart failure. Dis. Model. Mech..

[59] Csonka C., Sárközy M., Pipicz M., Dux L., Csont T. (2015). Modulation of Hypercholesterolemia-Induced Oxidative/Nitrative Stress in the Heart. Oxidative Med. Cell. Longev..

[60] Papatheodorou L., Weiss N. (2007). Vascular Oxidant Stress and Inflammation in Hyperhomocysteinemia. Antioxid. Redox Signal..

[61] Newsholme P., Cruzat V.F., Keane K.N., Carlessi R., de Bittencourt P.I. (2016). Molecular mechanisms of ROS production and oxidative stress in diabetes. Biochem. J..

[62] Roberts C.K., Sindhu K.K. (2009). Oxidative stress and metabolic syndrome. Life Sci..

[63] Mishra M., de Geest B. (2020). High-Density Lipoprotein-Targeted Therapies for Heart Failure. Biomedicines.

[64] De Geest B., Mishra M. (2021). Role of high-density lipoproteins in cardioprotection and in reverse remodeling: Therapeutic implications. Biochim. et Biophys. Acta (BBA) Mol. Cell Biol. Lipids.

[65] Sengupta S., Wehbe C., Majors A.K., Ketterer M.E., DiBello P.M., Jacobsen D.W. (2001). Relative Roles of Albumin and Ceruloplasmin in the Formation of Homocystine, Homocysteine-Cysteine-mixed Disulfide, and Cystine in Circulation. J. Biol. Chem..

[66] Heinecke J.W., Rosen H., Suzuki L.A., Chait A. (1987). The role of sulfur-containing amino acids in superoxide production and modification of low density lipoprotein by arterial smooth muscle cells. J. Biol. Chem..

[67] Muthuramu I., Singh N., Amin R., Nefyodova E., Debasse M., Van Horenbeeck I., Jacobs F., De Geest B. (2015). Selective homocysteine-lowering gene transfer attenuates pressure overload-induced cardiomyopathy via reduced oxidative stress. J. Mol. Med..

[68] Muthuramu I., Jacobs F., Singh N., Gordts S.C., De Geest B. (2013). Selective Homocysteine Lowering Gene Transfer Improves Infarct Healing, Attenuates Remodelling, and Enhances Diastolic Function after Myocardial Infarction in Mice. PLoS ONE.

[69] Duarte M.M., Moresco R.N., Duarte T., Santi A., Bagatini M.D., Da Cruz I.B., Schetinger M.R., Loro V.L. (2010). Oxidative stress in hypercholesterolemia and its association with Ala16Val superoxide dismutase gene polymorphism. Clin. Biochem..

[70] Farnaghi S., Prasadam I., Cai G., Friis T., Du Z., Crawford R., Mao X., Xiao Y. (2017). Protective effects of mitochondria-targeted antioxidants and statins on cholesterol-induced osteoarthritis. FASEB J..

[71] Oliveira C.H., Cosso G.R., Alberici C.L., Maciel N.E., Salerno G.A., Dorighello G.G., Velho A.J., De Faria E.C., Vercesi A.E. (2005). Oxidative stress in atherosclerosis-prone mouse is due to low antioxidant capacity of mitochondria. FASEB J..

[72] Muthuramu I., Mishra M., Aboumsallem J.P., Postnov A., Gheysens O., De Geest B. (2019). Cholesterol lowering attenuates pressure overload-induced heart failure in mice with mild hypercholesterolemia. Aging.

[73] Muthuramu I., Amin R., Postnov A., Mishra M., Aboumsallem J.P., Dresselaers T., Himmelreich U., Van Veldhoven P.P., Gheysens O., Jacobs F. (2017). Cholesterol-Lowering Gene Therapy Counteracts the Development of Non-ischemic Cardiomyopathy in Mice. Mol. Ther..

[74] Van Craeyveld E., Jacobs F., Gordts S.C., De Geest B. (2011). Low-density lipoprotein receptor gene transfer in hypercholesterolemic mice improves cardiac function after myocardial infarction. Gene Ther..

[75] Marseglia L., Manti S., D’Angelo G., Nicotera A.G., Parisi E., Di Rosa G., Gitto E., Arrigo T. (2014). Oxidative Stress in Obesity: A Critical Component in Human Diseases. Int. J. Mol. Sci..

[76] Aboumsallem J.P., Muthuramu I., Mishra M., De Geest B. (2019). Cholesterol-Lowering Gene Therapy Prevents Heart Failure with Preserved Ejection Fraction in Obese Type 2 Diabetic Mice. Int. J. Mol. Sci..

[77] Soran H., Schofield J.D., Durrington P.N. (2015). Antioxidant properties of HDL. Front. Pharmacol..

[78] Mackness M., Arrol S., Abbott C., Durrington P. (1993). Protection of low-density lipoprotein against oxidative modification by high-density lipoprotein associated paraoxonase. Atheroscler..

[79] De Geest B., Stengel D., Landeloos M., Lox M., Le Gat L., Collen D., Holvoet P., Ninio E. (2000). Effect of overexpression of human apo A-I in C57BL/6 and C57BL/6 apo E-deficient mice on 2 lipoprotein-associated enzymes, platelet-activating factor acetylhydrolase and paraoxonase. Comparison of adenovirus-mediated human apo A-I gene transfer and human apo A-I transgenesis. Arter. Thromb Vasc. Biol..

[80] Tabet F., Rye K.-A. (2009). High-density lipoproteins, inflammation and oxidative stress. Clin. Sci..

[81] Van Linthout S., Spillmann F., Riad A., Trimpert C., Lievens J., Meloni M., Escher F., Filenberg E., Demir O., Li J. (2008). Human Apolipoprotein A-I Gene Transfer Reduces the Development of Experimental Diabetic Cardiomyopathy. Circulation.

[82] Van Linthout S., Spillmann F., Lorenz M., Meloni M., Jacobs F., Egorova M., Stangl V., De Geest B., Schultheiss H.-P., Tschope C. (2009). Vascular-Protective Effects of High-Density Lipoprotein Include the Downregulation of the Angiotensin II Type 1 Receptor. Hypertension.

[83] Wenzel P., Munzel T. (2009). From menace to marvel: High-density lipoprotein prevents endothelial nitric oxide synthase uncoupling in diabetes mellitus by angiotensin II type 1 receptor downregulation. Hypertension.

[84] Amin R., Muthuramu I., Aboumsallem J.P., Mishra M., Jacobs F., De Geest B. (2017). Selective HDL-Raising Human Apo A-I Gene Therapy Counteracts Cardiac Hypertrophy, Reduces Myocardial Fibrosis, and Improves Cardiac Function in Mice with Chronic Pressure Overload. Int. J. Mol. Sci..

[85] Christison J., Karjalainen A., Brauman J., Bygrave F., Stocker R. (1996). Rapid reduction and removal of HDL- but not LDL-associated cholesteryl ester hydroperoxides by rat liver perfused in situ. Biochem. J..

[86] Van Eck M., Hoekstra M., Hildebrand R.B., Yaong Y., Stengel D., Kruijt J.K., Sattler W., Tietge U.J., Ninio E., Van Berkel T.J. (2007). Increased Oxidative Stress in Scavenger Receptor BI Knockout Mice with Dysfunctional HDL. Arter. Thromb. Vasc. Biol..

[87] Rigotti A., Trigatti B.L., Penman M., Rayburn H., Herz J., Krieger M. (1997). A targeted mutation in the murine gene encoding the high density lipoprotein (HDL) receptor scavenger receptor class B type I reveals its key role in HDL metabolism. Proc. Natl. Acad. Sci. USA.

[88] Linton M.F., Tao H., Linton E.F., Yancey P.G. (2017). SR-BI: A Multifunctional Receptor in Cholesterol Homeostasis and Atherosclerosis. Trends Endocrinol. Metab..

[89] Muthuramu I., Amin R., Aboumsallem J.P., Mishra M., Robinson E.L., De Geest B. (2018). Hepatocyte-Specific SR-BI Gene Transfer Corrects Cardiac Dysfunction in Scarb1 -Deficient Mice and Improves Pressure Overload-Induced Cardiomyopathy. Arter. Thromb. Vasc. Biol..

[90] Mishra M., Muthuramu I., de Geest B. (2019). HDL dysfunction, function, and heart failure. Aging.

[91] O’Brien J., Hayder H., Zayed Y., Peng C. (2018). Overview of MicroRNA Biogenesis, Mechanisms of Actions, and Circulation. Front. Endocrinol..

[92] Alles J., Fehlmann T., Fischer U., Backes C., Galata V., Minet M., Hart M., Abu-Halima M., Grässer F.A., Lenhof H.-P. (2019). An estimate of the total number of true human miRNAs. Nucleic Acids Res..

[93] Latronico M.V.G., Condorelli G. (2009). MicroRNAs and cardiac pathology. Nat. Rev. Cardiol..

[94] Kura B., Bacova B.S., Kalocayova B., Sykora M., Slezak J. (2020). Oxidative Stress-Responsive MicroRNAs in Heart Injury. Int. J. Mol. Sci..

[95] Ali Sheikh M.S., Salma U., Zhang B., Chen J., Zhuang J., Ping Z. (2016). Diagnostic, Prognostic, and Therapeutic Value of Circulating miRNAs in Heart Failure Patients Associated with Oxidative Stress. Oxid Med. Cell. Longev..

[96] Wang Z. (2011). The Guideline of the Design and Validation of MiRNA Mimics. Methods Mol. Biol..

[97] Ebert M.S., Sharp P.A. (2010). MicroRNA sponges: Progress and possibilities. RNA.

[98] Wang X.-W., Zhang C., Lee K.-C., He X.-J., Lü Z.-Q., Huang C., Wu Q.-C. (2017). Adenovirus-Mediated Gene Transfer of microRNA-21 Sponge Inhibits Neointimal Hyperplasia in Rat Vein Grafts. Int. J. Biol. Sci..

[99] Gentner B., Schira G., Giustacchini A., Amendola M., Brown B.D., Ponzoni M., Naldini L. (2008). Stable knockdown of microRNA in vivo by lentiviral vectors. Nat. Chem. Biol..

[100] Simms C., Zaher H.S. (2016). Quality control of chemically damaged RNA. Cell. Mol. Life Sci..

[101] Robson A. (2020). Oxidation of miRNAs by ROS leads to cardiac hypertrophy. Nat. Rev. Cardiol..

[102] De Geest B., Mishra M. (2021). Doxorubicin-induced cardiomyopathy: TERT gets to the heart of the matter. Mol. Ther..

[103] Kang Y.J., Chen Y., Epstein P. (1996). Suppression of Doxorubicin Cardiotoxicity by Overexpression of Catalase in the Heart of Transgenic Mice. J. Biol. Chem..

[104] Johnson J.A., Johnson D.A., Kraft A.D., Calkins M.J., Jakel R.J., Vargas M.R., Chen P.C. (2008). The Nrf2-ARE pathway: An indicator and modulator of oxidative stress in neurodegeneration. Ann. N. Y. Acad. Sci..

[105] Zhang W.-B., Lai X., Guo X.-F. (2021). Activation of Nrf2 by miR-152 Inhibits Doxorubicin-Induced Cardiotoxicity via Attenuation of Oxidative Stress, Inflammation, and Apoptosis. Oxidative Med. Cell. Longev..

[106] Itoh K., Tong K.I., Yamamoto M. (2004). Molecular mechanism activating nrf2–keap1 pathway in regulation of adaptive response to electrophiles. Free Radic. Biol. Med..

[107] Masson S., Batkai S., Beermann J., Bär C., Pfanne A., Thum S., Magnoli M., Balconi G., Nicolosi G.L., Tavazzi L. (2018). Circulating microRNA-132 levels improve risk prediction for heart failure hospitalization in patients with chronic heart failure. Eur. J. Heart Fail..

[108] Zhang L., Huang D., Wang Q., Shen D., Wang Y., Chen B., Zhang J., Gai L. (2014). MiR-132 Inhibits Expression of SIRT1 and Induces Pro-inflammatory Processes of Vascular Endothelial Inflammation through Blockade of the SREBP-1c Metabolic Pathway. Cardiovasc. Drugs Ther..

[109] Strum J.C., Johnson J.H., Ward J., Xie H., Feild J., Hester A., Alford A., Waters K.M. (2009). MicroRNA 132 Regulates Nutritional Stress-Induced Chemokine Production through Repression of SirT. Mol. Endocrinol..

[110] Singh C.K., Chhabra G., Ndiaye M.A., Garcia-Peterson L.M., Mack N.J., Ahmad N. (2018). The Role of Sirtuins in Antioxidant and Redox Signaling. Antioxid. Redox Signal..

[111] Chen B., Zang W., Wang J., Huang Y., He Y., Yan L., Liu J., Zheng W. (2015). The chemical biology of sirtuins. Chem. Soc. Rev..

[112] Nakagawa T., Guarente L. (2011). Sirtuins at a glance. J. Cell Sci..

[113] Huang K., Gao X., Wei W. (2017). The crosstalk between Sirt1 and Keap1/Nrf2/ARE antioxidative pathway forms a positive feedback loop to inhibit FN and TGF-beta1 expressions in rat glomerular mesangial cells. Exp. Cell Res..

[114] Gurd B.J. (2011). Deacetylation of PGC-1α by SIRT1: Importance for skeletal muscle function and exercise-induced mitochondrial biogenesis. Appl. Physiol. Nutr. Metab..

[115] Rius-Pérez S., Torres-Cuevas I., Millán I., Ortega Á.L., Pérez S. (2020). PGC-1alpha, Inflammation, and Oxidative Stress: An Integrative View in Metabolism. Oxid. Med. Cell. Longev..

[116] Ucar A., Gupta S.K., Fiedler J., Erikci E., Kardasinski M., Batkai S., Dangwal S., Kumarswamy R., Bang C., Holzmann A. (2012). The miRNA-212/132 family regulates both cardiac hypertrophy and cardiomyocyte autophagy. Nat. Commun..

[117] Kops G., Dansen T.B., Polderman P.E., Saarloos I., Wirtz K.W.A., Coffer P.J., Huang T.-T., Bos J.L., Medema R., Burgering B. (2002). Forkhead transcription factor FOXO3a protects quiescent cells from oxidative stress. Nat. Cell Biol..

[118] Olmos Y., Valle I., Borniquel S., Tierrez A., Soria E., Lamas S., Monsalve M. (2009). Mutual Dependence of Foxo3a and PGC-1α in the Induction of Oxidative Stress Genes. J. Biol. Chem..

[119] Condorelli G., Ferrante G. (2021). MicroRNA-132 Inhibition Prevents Myocardial Hypertrophy and Heart Failure in Pigs: Making Sense Out of Antisense. J. Am. Coll. Cardiol..

[120] Zhou Y., Li K., Liu L., Li S. (2020). MicroRNA-132 promotes oxidative stress-induced pyroptosis by targeting sirtuin 1 in myocardial ischaemia-reperfusion injury. Int. J. Mol. Med..

[121] Batkai S., Genschel C., Viereck J., Rump S., Bär C., Borchert T., Traxler D., Riesenhuber M., Spannbauer A., Lukovic D. (2021). CDR132L improves systolic and diastolic function in a large animal model of chronic heart failure. Eur. Heart J..

[122] Hinkel R., Batkai S., Bähr A., Bozoglu T., Straub S., Borchert T., Viereck J., Howe A., Hornaschewitz N., Oberberger L. (2021). AntimiR-132 Attenuates Myocardial Hypertrophy in an Animal Model of Percutaneous Aortic Constriction. J. Am. Coll. Cardiol..

[123] Täubel J., Hauke W., Rump S., Viereck J., Batkai S., Poetzsch J., Rode L., Weigt H., Genschel C., Lorch U. (2021). Novel antisense therapy targeting microRNA-132 in patients with heart failure: Results of a first-in-human Phase 1b randomized, double-blind, placebo-controlled study. Eur. Heart J..

[124] Jones N., Hobbs F.R., Taylor C.J. (2017). Prognosis following a diagnosis of heart failure and the role of primary care: A review of the literature. BJGP Open.

